# Local and metastatic curative radiotherapy in patients with de novo oligometastatic prostate cancer

**DOI:** 10.1038/s41598-020-74562-3

**Published:** 2020-10-15

**Authors:** C. Reverberi, M. Massaro, M. F. Osti, D. Anzellini, L. Marinelli, A. Montalto, V. De Sanctis, M. Valeriani

**Affiliations:** Radiotherapy Department, Sant’Andrea Hospital, La Sapienza II, University of Rome, Rome, Italy

**Keywords:** Cancer, Prostate cancer

## Abstract

The aim of this observational study is to investigate whether local consolidative treatment delivered to the primary site and metastatic tumour burden may add survival benefit to de novo oligometastatic prostate cancer (Oligo-PCa) patients. We retrospectively reviewed all Oligo-PCa patients treated with radiotherapy to the primary tumor sites and metastatic tumor burden at our institution between March 2010 and June 2019. All patients having ≤ 5 metastases involving nodes and/or bones, loco-regional and/or extra-pelvic sites, were included. Most of the patients had started androgen deprivation therapy with or without docetaxel as standard of care before radiotherapy. The Kaplan Meier analysis was performed to estimate survival outcomes. The univariate analysis tested possible prognostic factors increasing the rate of biochemical relapse. We analysed 37 Oligo-PCa patients. Twenty-eight (75.7%) had loco-regional metastases, in 9 patients (24.3%) the metastatic tumour burden was extra-pelvic. Nineteen (51.4%) had bone metastases, 21 (56.8%) nodal involvement and 7 (18.9%) both. Twenty (54.1%) had a single metastasis. The median follow-up was 55.5 months. The median overall survival (OS) was 68.8 months, the 2- and 5-year OS rates were 96.9% and 65.4%. The median biochemical relapse free survival (b-RFS) was 58 months and the 2- and 5-year b-RFS rates were 73.3% and 39.3%. The 2- and 5-year local relapse free survival rates were 93.9% and 83.7%. On the univariate analysis post-treatment PSA level ≤ 1 ng/ml was significantly related with the b-RFS (*p* = 0.004). Curative approach in Oligo-PCa patients involving both the primary tumor and metastatic sites may be feasible and well tolerate. Many patients presented longer survival and PSA at first follow-up was the most important prognostic factor. Further trials are needed to confirm our results and to evaluate if patients with PSA at first follow-up > 1 ng/ml may benefit from further treatments.

## Introduction

Currently, the oligo-metastatic disease is defined as synchronous or metachronous 3–5 metastases, which may include the presence of the primary tumor. Although there is a continuous technological development in more sensible diagnostic devices, nowadays doesn’t exist a diagnostic tool able to detect the presence of micro-metastatic disease. For this reason, the correct definition of oligometastatic status could be an intermediate state between localized disease and aggressive metastatic cancer. Preliminary finding supports the hypothesis of a true oligometastatic biology, different from a potentially metastatic cancer in which the limited number of lesions is the initial manifestation of a more widespread process. The ability to distinguish these disease states is crucial for identify those patients who had better prognostic outcome and may benefit from more aggressive treatment options compared to patients with metastatic disease^1^. The term oligometastatic prostate cancer (Oligo-PCa) describes the synchronous diagnosis of metastases and untreated primary in the castration-naïve state. Singh et al^2^ investigated prostate cancer patients survival as a function of the number of metastases: they found that men with ≤ 5 metastases had similar survival to those without metastases and significantly better survival compared to patients with more than 5 lesions (*p* = 0.02). There is no consensus on the optimal treatment strategy, lacked prospective randomized trial exploring the benefit of adding radical ablative treatment to systemic treatment options^3^. Several groups have investigated the benefit of adding a metastases-direct therapy to standard systemic treatment^4^. The Italian Association of Radiotherapy and Clinical Oncology (AIRO) has published a consensus agreement on Stereotactic Body Radiation Therapy (SBRT) as Local Consolidative Treatment (LCT) in oligometastatic PCa^5^: in the setting of synchronous oligometastatic PCa, there are proved benefits by treating with curative intent both the primary and the metastatic tumour burden with SBRT followed by systemic therapy.

The aim of this observational study is to investigate whether local consolidative treatment delivered to both the primary and metastatic tumour burden may add survival benefit on oligometastatic PCa patients.

## Materials and methods

All methods were carried out in accordance with relevant guidelines and regulations and the study was approved by the internal review board. Informed consent was obtained from all subjects. Ethics Committee of Sapienza University has approved the study. We retrospectively reviewed all oligometastatic prostate cancer patients, radiotherapy naïve, treated at our institution between March 2010 and June 2019. Patients’ selection criteria were: age ≥ 18 years, adenocarcinoma of the prostate histologically proved, previously untreated with radiotherapy, with oligometastatic disease (defined as less than 5 metastases) treated at our institution with radiotherapy to the primary tumour site and to the metastatic tumour burden at the same time. Were included patients who developed loco-regional and/or extra-pelvic disease, involving bone and/or node. Prior radical prostatectomy was not an exclusion criteria. Most of the patients got androgen deprivation therapy (ADT) with or without docetaxel as standard of care.

At our hospital the metastatic burden was staged by whole body choline positron emission tomography-computed tomography (PET-CT). When indicated a pelvic Magnetic Resonance Imaging (MRI) was performed. The PSA value was registered before starting EBRT, 1 month after treatment and during the follow-up period (FUP): it is considered the most important marker for disease progression.

A Computed Tomography (CT) scan without contrast enhancement was completed to provide an accurate delineation of the clinical target volumes (CTV). The CTV of the primary tumour included different volumes for prostate, seminal vesicles and pelvic nodes. A separate CTV was drawn for the metastatic sites. The planning target volume (PTV) was the CTV expanded by 0.5 cm in all directions.

Radiotherapy was delivered daily in 5 weeks, the median total dose to the pelvis was 45 Gy (1.8 Gy/fraction), it ranged between 55 and 68.75 Gy (2.2–2.75 Gy/fraction) for seminal vesicles and 68.75 Gy (2.75 Gy/fraction) for the prostate. The total dose to the metastatic burden was different according to the site and the size of the lesion implicated. For bone metastases the dose commonly used was 45–55 Gy/25 fractions, while loco-regional nodal metastases were usually treated with 55–60 Gy/25 fractions. Extra-pelvic metastases were more commonly treated with SBRT in 1–5 fractions.

Patients were followed up every 3 months during the first 6 months after EBRT, and then every 6 months. The FUP has included routine PSA analysis and clinical evaluation. The biochemical PSA relapse was defined as an increase of the PSA level by 2 ng/ml above the nadir value (after EBRT) or as values > 0.2 ng/ml following radical prostatectomy in accordance with the European Association of Urology (EAU) guidelines^6^. If the PSA level was rising, further scans were required to assess if there was a macroscopic progression either. Acute and late toxicity events were investigated and scored according to the Common Terminology Criteria for Adverse Events v4.0 (CTCAE).

The statistical analysis was performed by using SPSS vv25. The ROC curve was applied to classify the PSA level pre- and post-radiotherapy and to find out a threshold setting for biochemical failure. The Area Under the Curve (AUC) represents the measure of separability. Ideally, an excellent model has AUC near to 1, a value close to 0.5 means model has no class separation capacity. We accept 0.8 as a value able to predict a significant cut off.

The Kaplan Meier analysis was performed for assess the median overall survival (OS) defined as the time intercourse from EBRT to death for any causes or the last follow-up, the biochemical Relapse Free Survival (b-RFS) defined as a rising PSA level compared to nadir, the local–regional relapse free survival (LRFS) is defined as the progression within the radiotherapy field, the radiological progression-free survival (r-PFS) is defined as the progression disease in different sites.

Univariate analysis, was used to correlate OS, b-RFS, LRFS and r-PFS to possible prognostic factors: age, Gleason Score, PSA level at diagnosis, PSA level post-EBRT, number of metastases, number of site of metastases. A p-value ≤ 0.05 was considered significant.

## Results

This observational study analysed 37 oligometastatic prostatic cancer patients treated at S. Andrea Hospital in Rome (Italy) between March 2010 and June 2019. Patients and tumour characteristics are displayed in Table 1. The median age at diagnosis was 70 years (range 53–83), the median PSA level at diagnosis was 22.4 ng/ml (range 4.1–685) and 57% of them had a Gleason Score (GS) ≥ 8. Thirty-six (97.3%) had Androgen Deprivation Therapy (ADT) and 1 (2.7%) patient received chemotherapy (Docetaxel) before starting radiotherapy. Six patients (16.2%) had surgery (radical prostatectomy) as first line treatment approach. Twenty-eight (75.7%) patients had pelvic metastases, the metastatic tumour burden was extra-pelvic in 9 (24.3%) patients. Nineteen (51.4%) had bone metastases and 21 (56.8%) had nodal involvement, 7 (18.9%) both. Twenty (54.1%) had a single metastasis. The median PSA level detected before starting EBRT was 1.63 ng/ml (range 0.04–132), the median PSA level 1 month after treatment dropped down to 0.19 ng/ml (range 0.01–32.41).

**Table 1:** Patients and tumour characteristics.

Number of patients	37patients
Age at primary tumour diagnosis	70 years (range 53–83)
≤ 70 years	19 (51%)
> 70 years	18 (49%)
Age at treatment time	73 years (range 56–89)
Gleason Score	
GS 5 (2 + 3), 6 (3 + 3)	5/37 (13%)
GS 7 (3 + 4), 7 (4 + 3)	11/37 (30%)
GS 8 (3 + 5), 8 (4 + 4), 9 (4 + 5), 9 (5 + 4), 10 (5 + 5)	21/37 (57%)
PSA level at diagnosis	22.4 ng/ml (range 4.1–685)
Systemic treatment	
Androgen deprivation therapy	36/37 (97.3%)
Chemotherapy (Docetaxel)	1/37 (2.7%)
PSA level pre-EBRT	1.63 ng/ml (range 0.04–132)
PSA level 1 month after EBRT	0.19 ng/ml (range 0.01–32.41)
Number of metastases/patient	
1 metastasis	20/37 (54.1%)
2 metastasis	12/37 (32.4%)
3 metastasis	3/37 (8.1%)
4 metastasis	2/37 (5.4%)
Extra-pelvic disease	9/37 (24.3%)
Loco-regional metastases	28/37 (75.7%)
Site of oligometastatic disease	
Bone metastases	19/37 (51.4%)
Nodal metastases	21/37 (56.8%)
Bone and nodal metastases	7/37 (18.9%)

**Figure 1:**
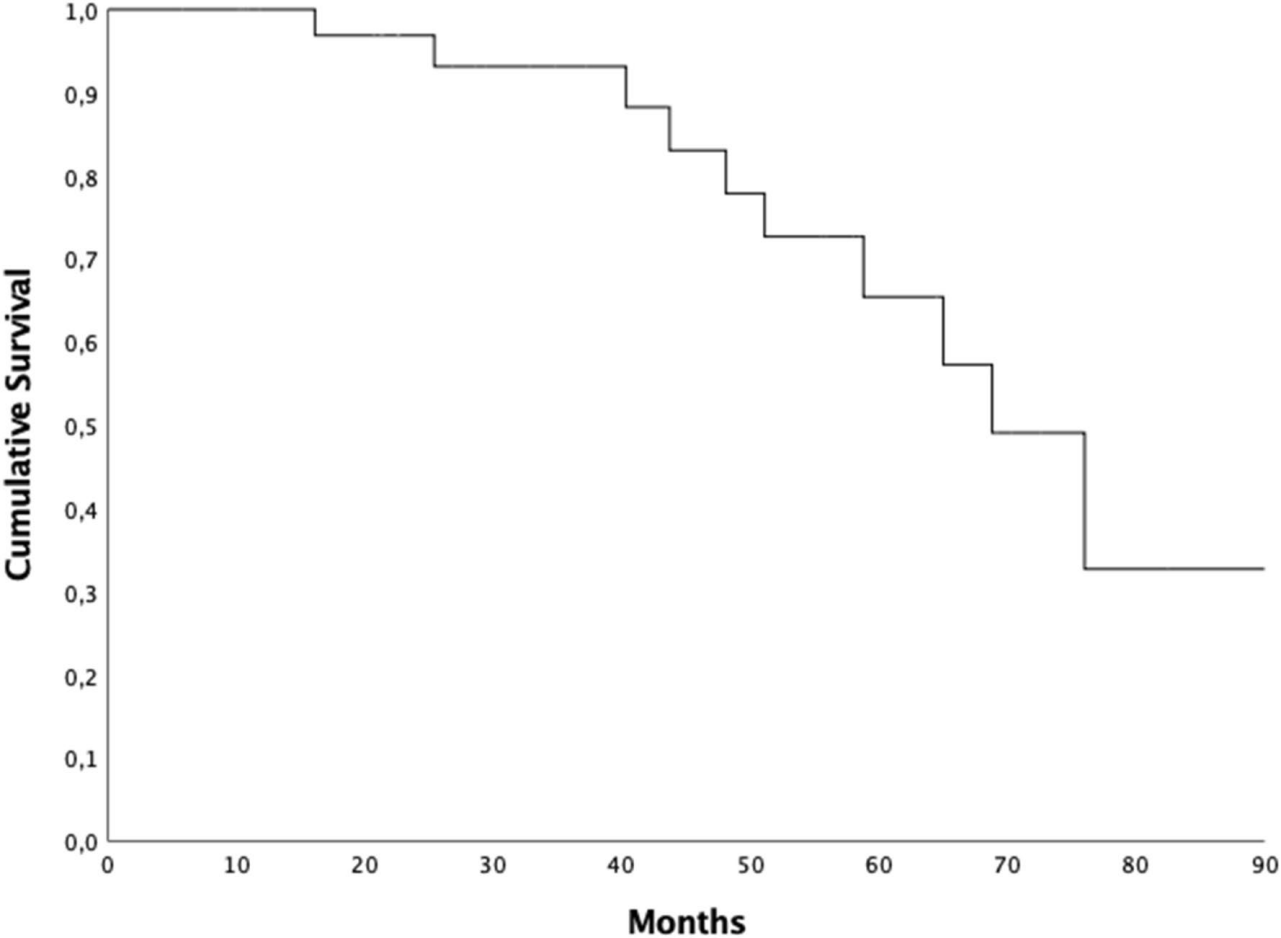
Kaplan-Meyer overall survival curve.

**Figure 2:**
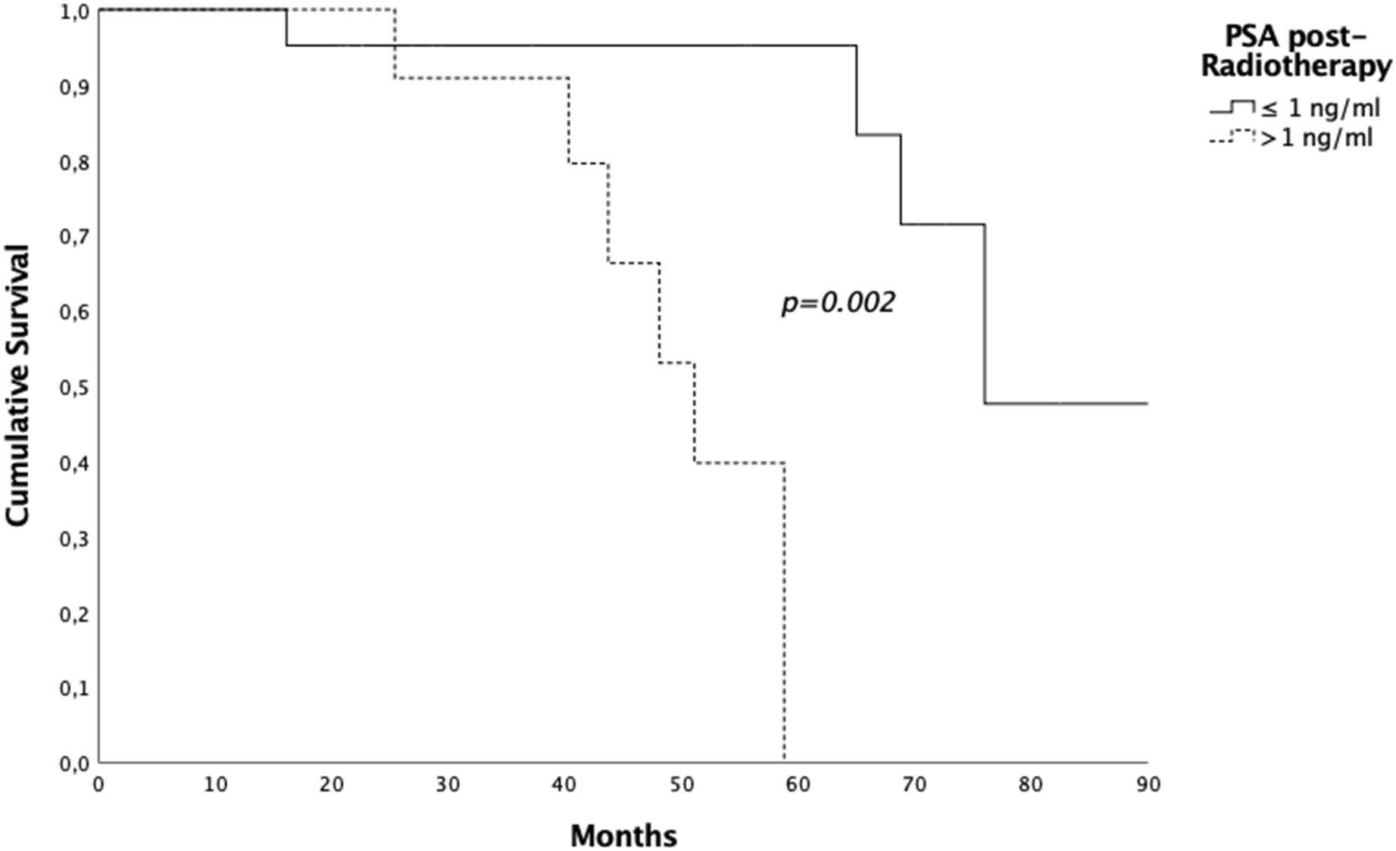
Kaplan-Meyer overall survival curve and PSA level post treatment.

In general, the median FUP was 55.5 months (range 5.8–90.1 months, 95% CI 25.4–85.6) and at the time of statistical analysis 26 (70.3%) patients were still alive. The median OS was 68.8 months (95%CI 57.32–80.28), the 2- and 5-years OS rates were 96.9% and 65.4% respectively (Fig. 1). Eleven (29.7%) patients passed away, 7 out of these died for disease. The median OS for patients with PSA level post EBRT ≤ 1 ng/ml was 76 months (95% CI 63.2–88.7), while it was 51.1 months (95%CI 41.5–60.7) when the PSA level post-EBRT was more than 1 ng/ml (*p* = *0.002*) (Fig. 2). The OS was not influenced by the number of metastases: median OS was 76 months (95% CI 59.6–92.3) for patients with single metastasis compared to 68.8 months (95% CI 42.1–95.4) for patients having more than one metastases (*p* = 0.62).

**Figure 3:**
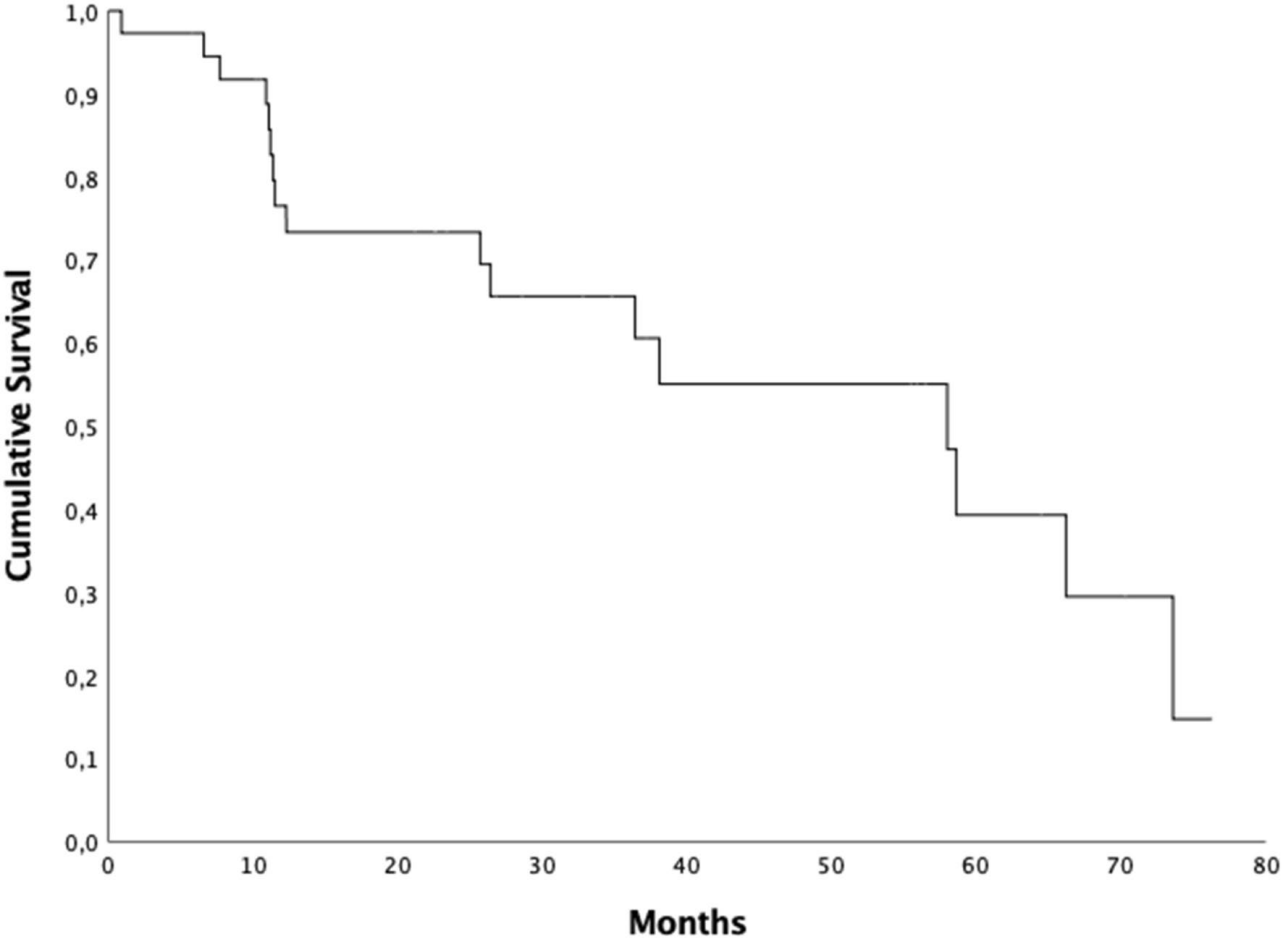
Kaplan Meyer biochemical relapse free survival curve.

**Figure 4:**
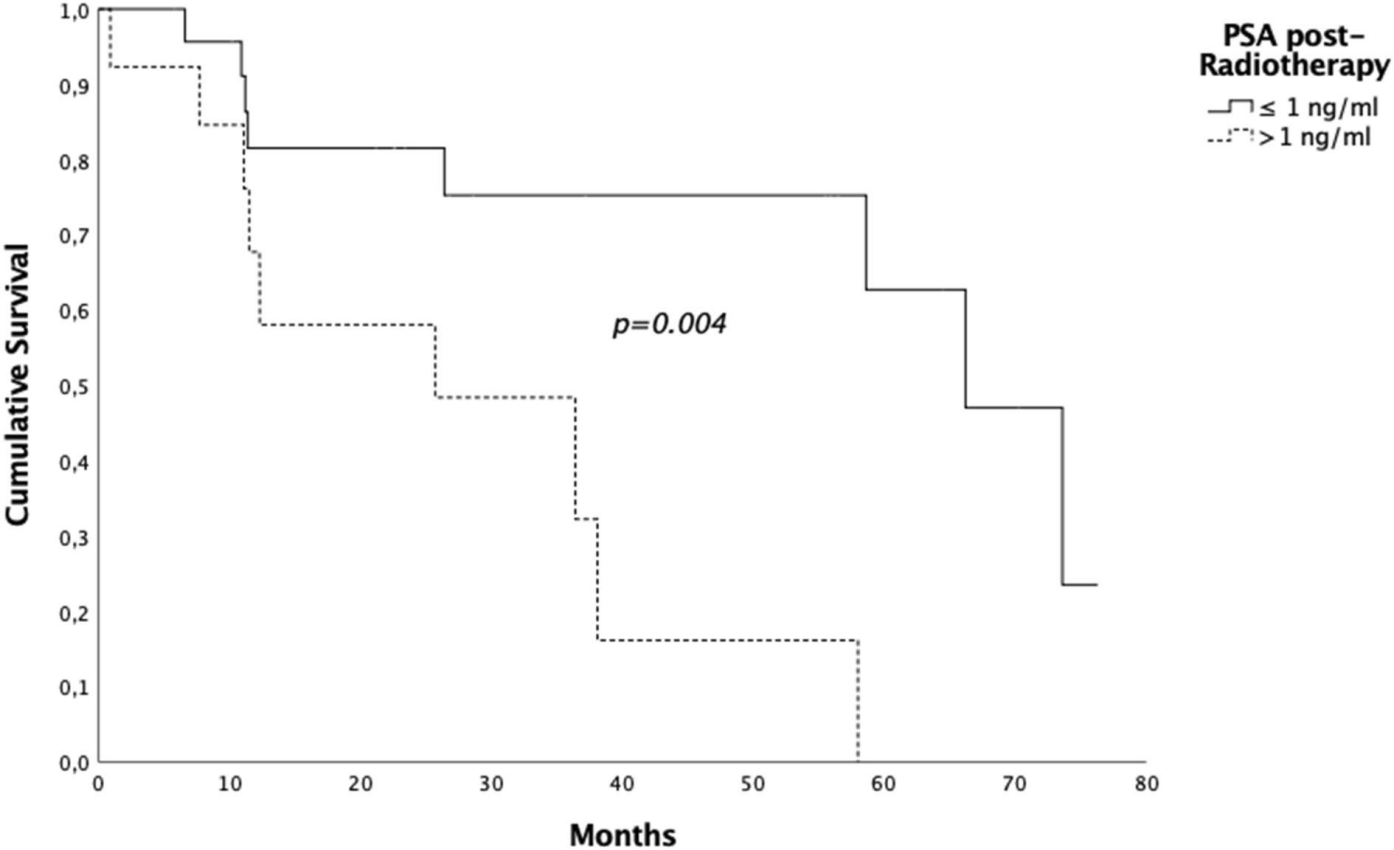
Five-year b-RFS rate and PSA level post treatment.

Seventeen (45.9%) patients had a pathological increased of PSA level during FUP, the median b-RFS was 58 months (95% CI 29.38–86.62) and the 2- and 5-years b-RFS rates were 73.3% and 39.3% respectively (Fig. 3). Patients having a PSA level 1 month after treatment ≤ 1 ng/ml had a median b-RFS of 66.2 months (95% CI 53.3–79.1) compared to 25.7 months (95%CI 0–53.2) in patients whom PSA level post-EBRT was > 1 ng/ml. The 5- year b-RFS rate in the first group of patients was 62.7% vs 16.71% (*p* = 0.004) (Fig. 4)*.* Patients having a PSA level at diagnosis < 10 ng/ml had a median b-RFS of 66.2 months (95% CI 0–0) compared to 38.1 months (95% CI 2.5–73.7) in patients whom PSA level at diagnosis was ≥ 10 ng/ml and the 5-year b-RFS rates were 64.3% and 30.3% in patients with PSA level at diagnosis < 10 ng/ml and PSA ≥ 10 ng/ml, respectively (*p* = 0.59).

The number of sites of metastases did not influence the b-RFS: the median value was 58 months (95%CI 14.1–101.8) for single site of metastasis and 58.6 months (95% CI 1–116.6) for patient with > 1 site involved (*p* = 0.22).

Four (10.8%) patients had a loco-regional relapse, the median LRFS was not reached and the 2- and 5-years LRFS rates were 93.9% and 83.7%.

Fifteen (40.5%) had distant progression, the median r-PFS was 60 months (95% CI 31.78–88.2), the 1-, 2- and 5-years r-PFS rates were 85.5%, 79.1% and 55.4%, respectively. Three patients (20%) had nodal relapse, 8 (53.3%) had bone progression, 3 (20%) had combined nodal and bone involvement, just 1 patient (6.7%) got lung metastases. Patients with a PSA level 1 month after treatment ≤ 1 ng/ml had a 5-year r-PFS of 69.9% vs 12.9% in patients with PSA level post-EBRT > 1 ng/ml (*p* = 0.01). Results were summarized in Table 2.

**Table 2:** Univariate analysis.

Variables	5-year OS %	*p* value	5-year bRFS %	*p* value	5-year LRFS %	*p* value	5-year r-PFS %	*p* value
Age		*0.91*		*0.85*		*0.28*		*0.49*
≤ 70 years	56.2		52.5		69.3		35.6	
> 70 years	72.7		34.4		93.3		56.3	
Gleason Score		*0.82*		*0.79*		*0.84*		*0.62*
≤ 7	58.3		36.7		78.6		45.2	
> 8	76.5		41.9		90.0		52.3	
PSA level at diagnosis		*0.51*		*0.59*		*0.99*		*0.31*
≤ 10 ng/ml	100.0		64.3		75.0		42.9	
> 10 ng/ml	57.9		30.3		81.0		43.6	
PSA level post-EBRT		***0.002***		***0.004***		*0.53*		***0.01***
≤ 1 ng/ml	100.0		62.7		88.0		69.9	
> 1 ng/ml	39.8		16.1		73.3		12.9	
Number of metastases		*0.62*		*0.74*		*0.10*		*0.8*
1	67.7		38.5		74.8		41.3	
> 1	61.9		43.5		100.0		58.0	
Number of site of metastases		*0.11*		*0.226*		*0.06*		*0.22*
1	80.0		43.4		71.9		46.6	
> 1	46.9		36.0		100.0		48.5	

OS, overall survival; bRFS; biochemical relapse free survival; LRFS, local relapse free survival; r-PFS, radiological progression-free survival; EBRT, external beam radiation therapy.

At progression time, the subsequent treatment involved a systemic approach with ADT or in castration-resistant PCa patients with a non-steroidal antiandrogen medication (Enzalutamide or Abiraterone) or the radioactive treatment (Radium, Ra 223-dichloride) or chemotherapy (Docetaxel). Nine patients were treated with radiotherapy to the new site of recurrence, 4 had re-irradiation for in-field relapse. When indicated, fractionated high intensity dose of radiotherapy (SABR) was the first choice of treatment, the most frequent dose/fractions used were: 27 Gy/3fr, 18 Gy/3fr and 56 Gy/8fr. Otherwise, fractionated radiotherapy for a total dose ranging between 20-45 Gy was delivered in 1.8–2 Gy per fraction.

Treatment related toxicities are indicated in Table 3. Overall, 18 (48.6%) patients experienced acute side effects. Fifteen (40.5%) had Genito-Urinary (GU) symptoms, most of whom were classified as Grade 1 (80%). Seven patients developed late GU toxicity, 5 of them (71.4%) previously referred acute GU toxicity. Five (13.5%) described acute Gastro-Intestinal (GI) toxicity after treatment, and just 1 (2.7%) patient had late GI symptoms.

**Table 3:** Treatment related toxicity.

Acute genito-urinary (GU)	15/37 (40.5%)
Grade 1	12/15 (80%)
Grade 2	3/15 (20%)
Acute gastro-intestinal (GI)	5/37 (13.5%)
Grade 1	4/5 (80%)
Grade 2	1/5 (20%)
Late GU (grade 1)	7/37 (18.9%)
Late GI (grade 1)	1/37 (2.7%)

GU, genito-urinary; GI, gastro-intestinal.

## Discussion

In 2019, the Advance Prostate Cancer Consensus Conference (APCCC) stated that in the setting of de novo oligometastatic prostate cancer, 54% of expert panellists voted for systemic therapy (ADT or docetaxel) plus treatment of the primary tumour and focal treatment of all lesions^3^. Our retrospective study with a median follow-up of 40.3 months, outlined that oligometastatic patients treated with metastasis direct-therapy and local therapy to the prostate had a median OS of 68.8 months (95%CI 57.32–80.28), and the 2- and 5-year OS rates were 96.9% and 65.4% respectively.

The STAMPEDE trial^7^ proved overall survival was improved in patients with low metastatic burden at baseline treated with ADT and radiotherapy to the prostate, over the control arm (HR 0·68, 95% CI 0·52–0·90; *p* = 0·007), the 3-year survival rate was 81% in patients received radiotherapy compared to 73% for patients treated with systemic therapy (ADT with or without docetaxel). Low metastatic burden disease is characterized, according to the CHAARTED^8^ definition, as an unlimited number of metastases provided they are confined to lymph nodes and the axial skeleton.

The STOPCAP meta-analysis of aggregate STAMPEDE and HORRAD data identified a 7% improvement in the 3-yr OS rate among men who had up to four bone metastases^9^.

According Gomez et al.^10^, the rational of adding a local consolidative treatment (LCT) to the standard systemic therapy, is that the tumour burden reduction could improve the efficacy of systemic treatment and minimized the risk of micro-metastatic disease. They randomized 49 patients to receive standard systemic therapy with or without LCT to the primary tumour site and metastases, the PFS was 11 months for the intervention arm vs 3.9 months in the control arm (HR 0.35, 90% CI: 0.18–0.66, *p* = 0.005).

Habl G. and colleagues^11^ retrospectively studied a small number of patients (15 patients) with oligometastatic prostate cancer having no more than 2 bone metastases, to receive curative SBRT (Biological Effective Dose- BED > 100 Gy) to the metastatic sites. They found the median b-RFS was 6.9 months (range 1.1–28.4 months), the 2 years LRFS was 100%. They described that curative SBRT given to the metastatic burden is safe and with high local control rate. Similarly, Jereczek-Fossa et al.^12^, treated 94 patients with nodal oligometastatic disease with SBRT, with a median total dose of 24 Gy (range 15-36 Gy) in 3 fractions (median BED 152 Gy). Most of the patients (94.7%) had 1 or 2 metastatic nodes. With a median FUP of 18.5 months, the 2-year local control and PFS rates were 84% and 30%, respectively; in patients who received concomitant ADT, the 2-years PFS rates was 49.4% vs. 22.6% in patients treated with SBRT alone. The in-field recurrence rate was very low and was observed in 9.7% of patients. Ost et al.^13^ retrospectively reviewed 119 patients with oligometastatic disease to bone, nodes or viscera treated with SBRT. The median follow-up was 36 months, the 3- and 5-years LRFS were 93% and 92%; and the 3- and 5-years distant relapse free interval rates were 31% and 15%, respectively. Patients treated with SBRT BED > 100 Gy had better LRFS (99% vs. 79%, *p* = 0.01).

In our analysis, the median b-RFS was 58 months (95% CI 29.38–86.62) and the 2- and 5-years b-RFS rates were 73.3% and 39.3%. Only 4 (10.8%) patients had a loco-regional relapse, the median LRFS was not reached and the 2- and 5-years LRFS rates were 93.9% and 83.7%. The better outcome achieved in our study can be explained by treating with curative intent both the prostate and metastatic burden. The PSA value at first follow-up was the most important prognostic factor whereas number and site of metastases didn’t influence survivals. This may imply the patients with PSA value at first follow-up > 1 ng/ml may beneficiate by an immediate start of systemic treatment, but further study are necessary to validate this hypothesis.

Some studies demonstrated that LCT can delay the beginning of palliative ADT in oligometastatic patients with controlled primary tumour, and limited the adverse effects induced by long-term ADT. Decaestecker K. et al.^14^ treated 50 patients with metachronous oligometastatic disease involving lymph nodes, bone, and viscera, with SBRT to a dose of 50 Gy in 10 fractions or 30 Gy in 3 fractions. ADT was initiated if patients had more than 3 synchronous metastases. With a median follow-up of 24 months, the local control rate was 100% and the median ADT-free survival was 25 months. Berkovic P. and colleagues^15^ studied 24 patients with 3 synchronous metastases (bone and/or lymph nodes) treated with SBRT to a dose of 50 Gy in 10 fractions. The median ADT-free survival was 38 months. The 2-year local control and clinical PFS were 100% and 42%, respectively.

It seems necessary having a good selection for oligometastatic prostate cancer patients who could benefit the most of a combined treatment approach (LCT and systemic treatment options). Franzese et al.^16^, hypothesised nodal involvement and castration- sensitive patients as possible prognostic factors for OS. They find out that OS was higher than 50% in patients with nodal involvement, and the distant progression free survival correlates with disease-free interval of 34 months cut off point. It is not well established the cut off PSA value prior treatment that could correlate with survival in oligometastatic patients, unless an increasing PSA value was related with a higher risk of progression (HR 1.01, 95% CI 1.00–1.02; *p* = 0.049). Decaestecker et al.^14^ illustrates the correlation between PSA doubling time and outcome: the median PFS was 12 months with doubling time < 3 months compared to 21 months for patients with longer doubling time prior LCT (*p* = 0.016). In our analysis we did not find a significant cut off for PSA value pre EBRT able to predict outcome, nevertheless, in the univariate analysis the PSA value post radiotherapy was statistically related with b-RFS (*p* = 0.05). Patients having a PSA level 1 month after treatment ≤ 1 ng/ml had a median b-RFS of 66.2 months (95% CI 53.3–79.1) compared to 25.7 months (95% CI 0–53.2) in patients whom PSA level post-EBRT was > 1 ng/ml.

Also, local therapies did not increase the rate of toxicity events. Iyengar and colleagues^17^ demonstrated that oligometastatic patients having Stereotactic ABlative Radiotherapy (SABR) prior to maintenance chemotherapy had a significant median PFS compared to patients who delayed SABR after progression over chemotherapy (9.7 vs 3.5 months, *p* = 0.01). There are more advantages in treating low tumour burden at hight growth rate over delayed SABR after chemotherapy where tumour speed rate is lower and total volume of treatment is bigger. We registered the rate of acute Gu toxicity was above 40%, with most of the patients referred Grade 1 toxicity (80%) and 13% of patients reported acute GI toxicity. No Grade 3 toxicity was reported. Overall, the rate of late toxicity was around 20%, all were Grade 1.

## Conclusion

De novo oligometastatic prostate cancer is a sub-group of patients with metastatic disease having better prognosis and who may benefit of more aggressive treatment. So far, data have demonstrated the benefit of combining systemic ADT with radiotherapy to the primary tumour in oligometastatic disease, and further studies have proved that metastasis-direct therapy improves local control and increase progression free survival. Nevertheless, most of these data came from observational study with small sample size and with a wide range definition of “oligometastatic disease”. The oligometastatic setting could be very heterogeneous, varying among de novo oligometastatic, recurrent oligometastatic, synchronous or metachronous, castration sensitive or castration resistant.

This is an observational study, involved a limited sample size of patients treated unevenly, aimed to show the effects of a combined curative treatment to both the primary and metastatic sites in de novo oligometastatic patients. We retrospectively reviewed patients with both bone and nodal metastases, without limiting the analysis to patients with loco-regional involvement. In conclusion, the purpose of this study is to underline possible benefit of curative radiotherapy in the setting of oligometastatic patients. PSA at first follow-up was the most important prognostic factor. Further trials are needed, trying to better define the oligometastatic disease in order to stratify patients by prognosis, to find the optimal dose of curative treatment in the era of SBRT, and the optimal timing with systemic therapy.
